# Risk management in digitalized educational environments: Teachers’ information security awareness levels

**DOI:** 10.3389/fpsyg.2022.986561

**Published:** 2022-09-08

**Authors:** Hamza Fatih Sapanca, Sezer Kanbul

**Affiliations:** Department of Computer Education and Instructional Technology, Near East University, Nicosia, Cyprus

**Keywords:** information security, awareness, teacher, risk management, sustainable society

## Abstract

With the spread of Information and Communication Technologies (ICT) tools and the Internet, Twenty first century technologies have significantly affected human life, and it has been desired to be obtained continuously. It has become challenging to protect information due to the increase in the methods by which malicious people can get information. As a result, it is crucial to determine people’s awareness levels by revealing the risks and threats to information security. In this context, a study was conducted to show the awareness levels of teachers who come after the family in raising conscious individuals in society. For this purpose, a quantitative research method was adopted for the problem and sub-problems that form the basis of the research. The survey model, one of the research designs used within the framework of the quantitative research method, was used. Information Security Awareness Scale was applied to 394 teachers, and according to the results obtained, it was determined that the information security awareness level of the teachers was moderate. According to the attacks and threats sub-dimension, which includes technical issues, it has been determined that the awareness levels of the teachers are at a medium level. The study results show that female teachers’ information security awareness levels are lower than male teachers. In comparison, the awareness levels of those who received information security awareness training and information technology teachers are higher.

## Introduction

Until the twentieth century, investments that required physical infrastructures such as land and factories were replaced by information from the twenty-first century (Couldry and Mejias, 2019). With the spread of computers, smart devices, and the Internet, twenty-first century technologies have penetrated almost all areas of human life, and as a result, information has become even more valuable and obtained its value (Yıldız Korkmaz and Atasoy, 2016; Grusho et al., 2018). The classic combination of companies, states, institutions, individuals, and societies in the twenty-first century is that they live in the information age and must keep up with the information age requirements (Lin, 2021).

As of 2022, 67.1% of the 7.91 billion population worldwide are mobile phone users, 62.5% are internet users, and 58.4% are social media users (Digital, 2022). This indicates that we are faced with a digitalized world, and as a result, data production has reached an incredible speed (Alacadağlı, 2019). It is essential to transform the produced data into information by making it functional (Fukuyama, 2018) and for society. A strong community is formed with the correct use of information (Park, 2017). In addition, various difficulties and social problems can be solved for the community (Sajidan et al., 2020). The sustainability of a strong community can be achieved with the correct use of technology (Çalış Duman, 2022). The proper use of technology means integrating society with artificial intelligence, networked control, the Internet of things, provision of services with technology, robots, and cyber security (Nagahara, 2019). In addition, European Union countries have drawn attention to the importance of cyber security for sustainable development, stating that cyber security is a basic need in a sustainable society (Sulich et al., 2021). Cyber security is crucial because it protects information (de Bruijn and Janssen, 2017).

With Information and Communication Technologies (ICT) tools, people can access information easily (Ghafir et al., 2018) and fast (Rahmatullah et al., 2022) at home, school, and the workplace (Ortaş, 2018). This situation has also increased the number of threats to information security (Jouini et al., 2014; Irmak and Baz, 2019). Human-made threats sometimes cause risks in information security breaches and occasionally natural disasters (Metalidou et al., 2014; Yaşar and Çakır, 2015). Human is the most crucial factor in information security (Evans et al., 2018, 2019). It is impossible to talk about the successful provision of information security without human beings (Colwill, 2009; Bostan and Şengül, 2018). The human factor is why many cyber-attacks on computers and systems are successful (Hughes-Lartey et al., 2021). The attacks on people sometimes result from the negligence, ignorance, and carelessness of the user (Yaşar and Çakır, 2015) and sometimes from users abusing their authority (Parsons et al., 2014; Ghafir et al., 2018). When the information security breach incidents experienced by world-renowned IT companies in recent years are examined in detail, it has been seen that the primary source of the problem is the lack of awareness of the employees on information, information technologies, and information security (Khando et al., 2021).

To continue education and training after the closure of schools during the pandemic, 18 million 241 thousand 881 students and 1 million 117 thousand teachers started the distance education process in schools affiliated with the Ministry of National Education in Turkey on March 23, 2020. The process was provided through the Education Information Network (EBA) platform. According to the 2021 global digitalization report, the EBA platform used in distance education is one of the most hit pages in Google searches (Özok and Tayiz, 2020). Technological tools are used intensively at all levels, from primary to high school (Özok and Tayiz, 2020). This is because digital media is moving extensive data around the Internet (Avcı and Oruç, 2020), causing concern for data security and increased risk (Lewandowski, 2019; Gökçearslan et al., 2021). Factors drivi Baryannis ng trouble are the disclosure of personal information, exposure to inappropriate online content, online security risks, and threats to the use of the Internet and smart devices (Kritzinger, 2017). It is necessary to eliminate anxiety to ensure the sustainability of digitalized educational environments and reduce the risks.

## Literature review

### Information security

Information security is the safe delivery of information to the recipient without being seized by unauthorized persons (Höne and Eloff, 2002; Dlamini et al., 2009). Its security must be ensured to protect the information (Baykara et al., 2013; Grusho et al., 2018). In this context, it is necessary to protect the privacy, security, and accessibility components that form the basis of information security (Tchernykh et al., 2019). Providing information security, minimizing risks to information security, widespread use of the Internet (Aslay, 2017), increasing the number of cyber-attack methods (Jang-Jaccard and Nepal, 2014), and establishing a legal basis by lawmakers (İhtiyaroğlu, 2020) have become mandatory (Henkoğlu and Yılmaz, 2013).

### Risk management

Risk is defined as “suffering loss,” while risk management is defined as “the process of carefully and in detail identifying and evaluating the risks that may occur while performing the work of institutions or businesses in advance and taking measures to eliminate or minimize risks” (TDK, 2022). From an information security perspective, the risk is when a threat exploits a vulnerability to damage information or data (Bubenko, 2007; ISO-ISO/IEC, 2008; Khidzir et al., 2010). In other words, the loss of confidentiality, integrity, and availability are the essential components of information security (Yeboah-Boateng, 2013). Risk management is a concept of increasing importance (Öznacar and Dagli, 2016) and can be defined as identifying potential risk (Spears and Barki, 2010) and reducing it to an acceptable level (Spears and Barki, 2010; Tummala and Schoenherr, 2011) and ensuring that it remains at this level. The definition of an acceptable level in risk management is not fully defined (Fan and Stevenson, 2018; Baryannis et al., 2019). Based on the definition, it can be stated that the acceptable level is the protection of the confidentiality, integrity, and accessibility components that form the basis of information security.

### Digitalized education environment

Today, with the rapid development of information technology, the internet and technological products are used to research information and share ideas (Mashhadi and Kargozari, 2011). As a result, the fact that information is accessible at any time regardless of space and time has transformed the existing educational environments through the innovative structure of the age (Tılıç, 2020). As a result of this transformation, access to learning and educational resources can be at any time and place.

### Information security in educational institutions

It is seen that attacks on educational institutions have increased with the transfer of the education and training environment to online environments due to the pandemic (Waldman, 2020; William, 2020; Levin, 2021). Attackers target schools due to the high number of people in the school and easier access to personal accounts (Richardson et al., 2020). In addition, on the cyber map published by Checkpoint, a world-renowned security product, educational institutions are the most targeted by attackers (Checkpoint, 2022). In this context, information security is essential in educational institutions to prevent attacks against teachers and students in online environments. Security risks can be eliminated by revealing teachers’ information security awareness levels (Al-Shehri, 2012; Bogart, 2012).

### Information security awareness

The term “information security awareness” implies that users in an organization are ideally aware of their commitment to their security mission (Siponen, 2000; Kajzer et al., 2014). Minimizing security-related risks with awareness and maximizing the effectiveness of security techniques and procedures (Hart et al., 2020) have an important place in increasing the protection of information and data (da Veiga et al., 2020). To create this awareness, finding users who have received information security awareness training is essential. These users are critical to reducing threats within the organization (al Awawdeh and Tubaishat, 2014). It is essential to increase their awareness and provide an educational environment to eliminate or minimize the vulnerabilities caused by the human factor in information security (Kim, 2014; Aldawood and Skinner, 2019; Avcı and Oruç, 2020).

### Teachers’ awareness

The sustainability of digitalized educational environments is essential in terms of the correct use of technology (Öznacar, 2018). Teachers are responsible for raising future citizens in a sustainable society (Baena-Morales et al., 2020). Teachers need to be trained on information security and many other issues. However, some studies on teachers have shown that the level of awareness is not good enough (Akgün and Topal, 2015; Al-Janabi and Al-Shourbaji, 2016). However, it has been noted that efforts to raise teachers’ awareness are limited to some institutional publications, announcements, and informative websites; thus, these attempts lack interaction with the target audience of teaching (Kadıoğlu, 2019).

### Hypotheses

The human factor is essential in managing information security properly (Yerby and Floyd KevinFloyd, 2018; Odiaga et al., 2020). Protecting information and data is possible by ensuring that users are aware of information security and thus minimizing potential risks (Da Veiga, 2019). People’s level of information security, which is seen as the weakest link of information security, is directly related to awareness (Cox et al., 2001; Rezgui and Marks, 2008; Vardal, 2009). While more than half of the world’s population uses computer technology and communication technologies, it is essential how aware people are of the risks they may face (Gümüş, 2007; Keser and Güldüren, 2015). In this way, ensuring information security and minimizing risks is possible by raising awareness of people and using technological equipment correctly (Puhakainen, 2006; Şahinaslan et al., 2009; Al-Shehri, 2012). Especially in the information society we live in, in the age of technology where every field is rapidly digitalized, the level of information security awareness of teachers should be revealed in terms of following the developments, informing the society about the developments, and preparing them for the future. In this context, teachers’ information security awareness levels have been determined in previous studies, and the effects of different variables have been revealed. In the study conducted by Canoğulları (2021), it was determined that the information security awareness levels of teachers were slightly above the middle level, and according to the gender and branch variables, the results were in favor of male teachers, and in the branch variable, results were obtained in favor of Information Technologies teachers. In the study by Keser and Yayla (2021), it was found that teachers’ information security awareness levels were high and male teachers had higher awareness levels than female teachers. According to the branch distribution, it was determined that the awareness levels of Information Technologies teachers were higher than in other branches. The fact that the awareness levels of teachers who received awareness training were higher than those who did not is one of the results of this study (Keser and Yayla, 2021). Odiaga et al. (2020) found that teachers had little or no knowledge about basic information security awareness practices, roles, threats, risks, and attacks. The study by Kiss (2019) determined that pre-school teachers’ information security awareness levels were low. According to the results obtained in the study by Karabatak and Karabatak (2019) on administrators working in schools, it was determined that administrators’ information security awareness levels were slightly above the middle level. The same study also found that the awareness levels of male administrators were higher than the awareness levels of female administrators (Karabatak and Karabatak, 2019). Considering these studies, it is seen that studies on teachers’ awareness determination are limited (Chou and Chou, 2016; Canoğulları, 2021).

In this study, the following hypotheses were put forward based on previous research by applying the information security awareness scale to determine the information security awareness levels of primary and secondary school teachers working city of Amasya in Turkey:

H1: Teachers’ information security awareness levels are at a medium level.

H2: Information security awareness levels of teachers differ according to gender.

H3: Information security awareness levels of teachers differ according to their training status.

H4: Information security awareness levels of teachers differ according to their branches.

## Methodology

### Research design

In this study, the survey model, one of the quantitative research designs, was used for the problem and sub-problems that form the basis of the research. The purpose of using this model (Wallen and Fraenkel, 2013), which is used to collect data on the opinions, attitudes, and behaviors of individuals on a subject and to reveal the general structure of these individuals on the subject, is to determine the information security awareness levels of teachers and to examine their awareness levels in detail in terms of different variables.

### Sample

Based on the study population determined within the scope of this study, a study group was formed with the convenience sampling technique (Yıldırım and Şimşek, 2008). The convenient sampling method was preferred because it is flexible in terms of time and economy. In addition, easy-to-reach participants were included because participation in the research is voluntary, and it accelerates the research (Yıldırım and Şimşek, 2008). Within the scope of the research, 394 primary and secondary school teachers were reached, and participation in the data collection tool was voluntary in city of Amasya in Turkey.

### Instrument

The “Information Security Awareness Scale” developed by Çetinkaya et al., 2017 was used to obtain data from the participants. The scale has a three-factor structure and consists of 48 items. In the development of the scale, exploratory factor analysis was conducted using a study group of 316 participants, and it was determined that it consisted of 48 items under three dimensions. Subsequently, confirmatory factor analysis was applied to 200 participants, and the structure was confirmed. The overall reliability coefficient of the scale is 0.98. As the overall score on the scale and the scores for sub-factors increase, participants’ Information Security Awareness increases. The scale score ranges are shown in Table 1.

**TABLE 1:** Information security awareness scale and the lowest, highest scores and level ranges.

	Questions	Lowest score	Highest score	Low	Medium	Normal	High
Color coding							
Information security awareness scale	1–48	48	240	48–96	97–144	145–192	193–240
**Sub-factors**							
General security	1–13	13	65	13–26	27–39	40–52	53–65
Attack and threats	14–30	17	85	17–34	35–51	52–68	69–85
Mobile devices, privacy and communication	31–48	18	90	18–36	19–54	55–72	73–90

Colors meaning: If it is blue, the teacher awareness’ level is low; If it is green, the teacher awareness’ level is medium; If it is yellow, the teacher awareness’ level is normal; If it is red, the teacher awareness’ level is high.

Data were collected in 3 months covering May and July 2021, after obtaining the necessary ethics committee permission to obtain the data. The data collection process was carried out meticulously to determine the awareness of information security, as teachers had to continue their classes online due to the mandatory closures and restrictions experienced during the pandemic process.

### Data analysis

The study used Kolmogorov-Smirnov (KS) and skewness-kurtosis coefficients to determine whether the data showed a normal distribution. Shapiro-Wilks is used when the group size is less than 50, and KS is used when it is more than 50 (Kim and Park, 2019). KS test and skewness-kurtosis coefficients showed that the data showed normal distribution. In this context, descriptive statistics were applied for the first hypothesis, an independent *t*-test for the second and third hypotheses, and one-way ANOVA test for the fourth hypothesis.

### Sample characteristics

According to the personal information obtained from the teachers, 52.3% of the teachers are female, and 47.7% are male. 26.1% stated that they received training, while 73.9% stated that they did not receive training. According to the branch distribution of the participants, 30.7% Classroom Teachers, 7.4% Pre-school, 4.8% Special Education, 4.6% Science, 5.8% Social Studies, 4.6% Religious Culture and Moral Knowledge, 7.1% Turkish, 5.8% English, 4.3% Information Technologies, 3.6% Physical Education, 4.3% Mathematics, 2.3% Turkish Language and Literature, 3.3% Technology Design, 4.1% Guidance, and 7.4% Vocational Branch teachers.

### Research limitations

The study is limited to 364 primary and secondary school teachers for 3 months between May and July 2021. The study obtained results with gender, information security training status, and branch variables.

## Results

Teachers’ information security awareness levels and score distribution by sub-factors is shown in Table 2. According to the Information Security Awareness Scale, teachers’ overall score average is 144.78+38.87, which is “medium.” The general security sub-factor score averages are 45.50+9.42, the attack and threats sub-factor score average are 43.40+15.91, and the mobile devices, privacy, and communication sub-factor score average is 55.88+16.61. The awareness level of general security and mobile devices, privacy, and communication sub-factors is “normal.” However, the level of the attacks and threats sub-factor was determined as “medium.” The averages of the items belonging to this factor are, respectively; Average score of “I know what hoax is” 2.52+1.13, average score of “I know how to deal with chain email” is 2.68+1.12, “Spyware (spyware) average score of 2.74+1.14, average score of” “I can tell if there is spyware on my computer” 2.43+1.09, average score of “I know how to prevent spyware from being installed on my computer” is 2.41+1, Average score of 1, “I know about security measures against identity theft” is 2.63+1.14, “I know what fake virus protection software is.” Average score of 2.59+1.13, average score of “I know what a Denial of Service (DoS) attack” is 2.26+1.07, average score of “I know what a phishing attack is” is 2.34+ Average score of 1.07, “I know what a social engineering attack is” 2.28+1.05, average score of “I know how to act to avoid being attacked by social engineering” is 2.29+1.04, “Cyberbullying (I know what cyberbullying is)” average score is 3+1.19, “I know how to protect myself against cyberbullying” average score is 2.83+1.2, “I know how to protect children against cyberbullying” average score is 2.82+1, 18, “I know the security measures to be taken against attacks that personal digital assistants (PDAs) may be exposed to” average score is 2.41+1.05, “I know what the active content used in web pages is for” average score is 2.49+ 1.06, “I know what cookies are used on web pages” her average score is 2.69+1.09.

**TABLE 2:** Teachers’ information security awareness levels and score distribution by sub-factors.

	N	Min	Max	$\bar{\text{x}}$	Sd	
Awareness levels	394	57.00	240.00	144.78	38.87	
General security	394	15.00	65.00	45.50	9.42	
Attack and threats	394	17.00	85.00	43.40	15.91	
Mobile devices, privacy, and communication	394	18.00	90.00	55.88	16.61	

Colors meaning: If it is green, the teacher awareness’ level is medium; If it is yellow, the teacher awareness’ level is normal.

Teachers’ information security awareness levels and sub-factors relations by gender is shown in Table 3. As a result of the independent *t*-test conducted to determine whether there is a significant difference between teachers’ information security awareness levels and sub-factors with gender, a significant difference was determined between teachers’ awareness levels and gender. Male teachers’ awareness levels are higher than female teachers. In addition, male teachers’ awareness levels are higher in sub-factors than female teachers’ awareness levels.

**TABLE 3:** Teachers’ information security awareness levels and sub-factors relations by gender.

	Gender	*n*	$\bar{\text{x}}$	*S*	*t*	*P*
Information security awareness levels	Female	206	136.45	38.11	−4.564	0.00*
	Male	188	153.92	37.73		
General security	Female	206	43.42	9.21	−4.702	0.00*
	Male	188	47.77	9.14		
Attack and threats	Female	206	40.32	15.18	−4.113	0.00*
	Male	188	46.79	16.05		
Mobile devices, privacy, and communication	Female	206	52.71	16.72	−4.038	0.00*
	Male	188	59.35	15.81		

“*” is mean shows that it is significant. *P* < 0.05.

Teachers’ information security awareness levels and sub-factors relations by receiving education on information security is shown in Table 4. As a result of the independent *t*-test conducted to determine whether there is a significant difference between teachers’ information security awareness levels and sub-factors and their status of receiving education on information security, information security awareness levels of teachers who received training on awareness are higher than those who did not.

**TABLE 4:** Teachers’ information security awareness levels and sub-factors relations by receiving education on information security.

	Education	n	$\bar{\text{x}}$	*S*	*t*	*P*
Information security awareness levels	Yes	103	168.2039	37.66242	7.609	0.00*
	No	291	136.5017	35.85982		
General security	Yes	103	51.0097	8.64382	7.356	0.00*
	No	291	43.5498	8.91493		
Attack and threats	Yes	103	52.2427	17.20890	6.937	0.00*
	No	291	40.2818	14.19821		
Mobile devices, privacy, and communication	Yes	103	64.9515	14.71919	6.810	0.00*
	No	291	52.6701	16.06885		

“*” is mean shows that it is significant. *P* < 0.05.

Teachers’ information security awareness levels and sub-factors relations by branch is shown in Table 5. As a result of the one-way ANOVA test conducted to determine the significant difference between teachers’ information security awareness levels and sub-factors and their branches, as a result of the data obtained, the information security awareness levels of information technology branch teachers are higher than other branch teachers.

**TABLE 5:** Teachers’ information security awareness levels and sub-factors relations by branch.

		n	$\bar{\text{x}}$	Sd	*F*	*p*	Differences
Information security awareness levels	Classroom teaching	121	139.78	39.41	5.877	0.000	Information Technologies Branch—All other branches
	Pre-school	29	134.28	36.14			
	Special education	19	138.32	45.01			
	Science	18	133.95	38.96			
	Social studies	23	151.48	36.1			
	Religion and moral knowledge	18	137.12	24.3			
	Turkish	28	134.97	30.36			
	English	23	155.87	32			
	Information technologies	17	212	32.37			
	Physical education	14	170.86	33.38			
	Maths	17	143.65	20.64			
	Turkish language and literature	9	131.45	42.97			
	Technology design	13	135.7	34.07			
	Counseling	16	147.13	28.88			
	Others	29	142.97	36.64			
General security	Classroom teaching	121	45.27	10.21	3.891	0.000	Information Technologies Branch—All other branches
	Pre-school	29	42.69	10.44			
	Special education	19	43.11	10.75			
	Science	18	44.5	8.71			
	Social studies	23	45.92	9.96			
	Religion and moral knowledge	18	46.12	5.34			
	Turkish	28	43.33	7.45			
	English	23	46.31	7.61			
	Information technologies	17	58.65	6.68			
	Physical education	14	52	6.54			
	Maths	17	43.42	5.49			
	Turkish language and literature	9	42.23	11.08			
	Technology design	13	42	8.4			
	Counseling	16	45.69	6.71			
	Others	29	45.11	8.27			
Attack and threats	Classroom teaching	121	42.03	15.27	6.342	0.000	Information Technologies Branch—All other branches
	Pre-school	29	39.18	13.04			
	Special education	19	40.32	17.32			
	Sciences	18	39.28	15.72			
	Social sciences	23	46.44	15.12			
	Religion and moral knowledge	18	36.73	12.77			
	Turkish	28	39.75	12.64			
	English	23	46.66	14.38			
	Information technologies	17	72.36	15.21			
	Physical education	14	52.58	15.28			
	Maths	17	44.06	10.73			
	Turkish language and literature	9	36.78	16.39			
	Technology design	13	39.77	15.74			
	Counseling	16	43.75	10.61			
	Others	29	42.45	15.03			
Mobile devices, privacy and communication	Classroom teaching	121	52.49	17.67	4.920	0.000	Information Technologies Branch - All Other Branches
	Pre-school	29	52.42	15.99			
	Special education	19	54.9	19.4			
	Sciences	18	50.17	17.34			
	Social sciences	23	59.14	13.97			
	Religion and moral knowledge	18	54.28	11.39			
	Turkish	28	51.9	14.27			
	English	23	62.92	12.65			
	Information technologies	17	81	11.7			
	Physical education	14	66.29	13.06			
	Maths	17	56.18	7.62			
	Turkish language and literature	9	52.45	18.52			
	Technology design	13	53.93	12.2			
	Counseling	16	57.69	15.4			
	Others	29	55.42	15.08			

## Discussion

Teachers were expected to use technology quickly during the pandemic, produce materials that would enable students to learn, and be executives that will enable students to learn (Rapanta et al., 2020). Due to the mandatory closures experienced during the pandemic, education took place in digital environments, and as a result, the learning process has become more digital (Frolova et al., 2020). However, due to the focus on the execution of the process, research on concepts such as safety and possible risk situations has not been revealed (Arina and Anatolie, 2021). In this context, providing safer educational environments and raising awareness of attacks on information security that teachers may encounter is essential in the sustainability and risk management of digitalized educational environments.

*H1: Teachers’ information security awareness levels are at a medium level.*

As a result of the findings, teachers’ information security awareness levels were determined as “moderate.” This result is in line with the result of Kubacka et al. (2021) during the pandemic process. Among the studies conducted before the pandemic, Canoğulları (2021) also found a moderate level of awareness in the study conducted for teachers. However, in the study by Keser and Yayla (2021), it was determined that teachers’ information security awareness levels were high. While awareness levels of general security, mobile devices, privacy, and communication factors from sub-factors were at a “normal” level, the awareness level of attacks and threats sub-factor was determined as a “medium” level. When the items belonging to the factor are examined, it is seen that it contains technical terms. Teachers need to be more aware of attacks and threats in this context. Similar results were obtained in the studies of Filippidis et al. (2018). The participants, whose awareness level was average, pointed out that they needed to improve their awareness of the technical details of information security and the tools used in this field (Filippidis et al., 2018). The awareness levels of teachers who educate the future generations must be even higher to protect educational institutions, which are, in the first place, the target of attackers. It has been determined that teachers do not have sufficient knowledge about cyber-attacks, information theft, social engineering attacks, and malware, which are increasing daily. This situation can be interpreted as inadequate security of information in educational institutions.

*H2: Information security awareness levels of teachers differ according to gender.*

A relationship was found between gender and information security awareness level, general security, mobile devices, privacy, and communication factors sub-dimensions. It has been determined that the awareness level of women is lower than men in both the general scores and sub-factors obtained from the scale. One of the similar results obtained in the study is that there is a strong link between gender and awareness and that men have higher awareness levels than women (Farooq et al., 2015; Karabatak and Karabatak, 2019; Canoğulları, 2021; Keser and Yayla, 2021). The reason why information security awareness levels are in favor of males can be interpreted as male teachers using ICT tools more (Gudmundsdottir and Hatlevik, 2018). In addition, when we look at the statistics on the use of the Internet and ICT tools in Turkey, it is clear that men use ICT tools more than women (Digital, 2022).

*H3: Information security awareness levels of teachers differ according to their training status.*

Many studies demonstrate the importance of information security education (Ahlan et al., 2015; Zwilling et al., 2020; Hwang et al., 2021; Khando et al., 2021; Taha and Dahabiyeh, 2021). In these studies, training for information security reveals the importance of training in raising conscious individuals to ensure information security, preventing attacks against end users, and raising awareness about attack types. The fact that those who receive awareness training have higher levels of information security reveals the importance of receiving training on information security.

*H4: Information security awareness levels of teachers differ according to their branches.*

A significant difference was found between the branches of the teachers and the information security awareness levels and the sub-dimensions of general security, mobile devices, privacy, and communication factors. The difference is in favor of the Information Technologies Branch. There are studies supporting this result in the literature (Canoğulları, 2021; Keser and Yayla, 2021). It can be thought that the awareness levels of Information Technologies teachers are higher because they closely follow the technology due to their professional definitions and have a better command of the terms about information security.

In summary, it is essential to continue education safely (Akcil and Bastas, 2021) to minimize the risks of possible attacks in digitalized educational environments. In this context, protecting information with the measures to be taken for information security in educational environments means ensuring risk management. Considering this situation, in this study, teachers’ information security awareness levels were determined, and the effect of different variables was examined. The results obtained were discussed and presented to the literature.

### Recommendations

Information security is a concept that is the responsibility of every individual user of ICT. Due to the increase in the methods of malicious people to obtain information, institutions and organizations should pay more attention to information security awareness training. Training on information security should be provided within a scope that includes all individuals from an early age. Efforts should be made to increase the tendencies of women toward information security by ensuring that they participate more in the process. Since information technology teachers closely follow the concepts related to technology and technological developments, it is recommended that they increase the associations of learning outcomes and in-class activities related to these concepts in educational environments.

## Data availability statement

The datasets presented in this study can be found in online repositories. The names of the repository/repositories and accession number (s) can be found in the article/supplementary material.

## Ethics statement

The studies involving human participants were reviewed and approved by the Ethical Committee Board of Near East University. The patients/participants provided their written informed consent to participate in this study.

## Author contributions

HS wrote the introduction, method, findings, discussion, conclusion, and suggestions in line with the opinions and suggestions of SK. As a result, this study has obtained a result of the approval and contributions of both authors.
